# Machine learning to predict the development of recurrent urinary tract infection related to single uropathogen, *Escherichia coli*

**DOI:** 10.1038/s41598-022-18920-3

**Published:** 2022-10-14

**Authors:** Shuen-Lin Jeng, Zi-Jing Huang, Deng-Chi Yang, Ching-Hao Teng, Ming-Cheng Wang

**Affiliations:** 1 Department of Statistics, Institute of Data Science, and Center for Innovative FinTech Business Models, National Cheng Kung University, Tainan, Taiwan; 2 Department of Statistics, National Cheng Kung University, Tainan, Taiwan; 3 Department of Geriatrics and Gerontology, National Cheng Kung University Hospital, College of Medicine, National Cheng Kung University, Tainan, Taiwan; 4 Institute of Molecular Medicine, College of Medicine, National Cheng Kung University, Tainan, Taiwan; 5 Institute of Basic Medical Sciences, College of Medicine, National Cheng Kung University, Tainan, Taiwan; 6 Center of Infectious Disease and Signaling Research, National Cheng Kung University, Tainan, Taiwan; 7 Division of Nephrology, Department of Internal Medicine, National Cheng Kung University Hospital, College of Medicine, National Cheng Kung University, Tainan, Taiwan

**Keywords:** Computational biology and bioinformatics, Diseases, Risk factors

## Abstract

Recurrent urinary tract infection (RUTI) can damage renal function and has impact on healthcare costs and patients’ quality of life. There were 2 stages for development of prediction models for RUTI. The first stage was a scenario in the clinical visit. The second stage was a scenario after hospitalization for urinary tract infection caused by *Escherichia coli.* Three machine learning models, logistic regression (LR), decision tree (DT), and random forest (RF) were built for the RUTI prediction. The RF model had higher prediction accuracy than LR and DT (0.700, 0.604, and 0.654 in stage 1, respectively; 0.709, 0.604, and 0.635 in stage 2, respectively). The decision rules constructed by the DT model could provide high classification accuracy (up to 0.92 in stage 1 and 0.94 in stage 2) in certain subgroup patients in different scenarios. In conclusion, this study provided validated machine learning models and RF could provide a better accuracy in predicting the development of single uropathogen (*E. coli*) RUTI. Both host and bacterial characteristics made important contribution to the development of RUTI in the prediction models in the 2 clinical scenarios, respectively. Based on the results, physicians could take action to prevent the development of RUTI.

## Introduction

Urinary tract infection (UTI) is one of the most common infectious diseases^1^. More than 30% of women will experience a subsequent infection within 12 months of resolution of the initial symptoms despite appropriate antibiotic therapy^2^. UTI and recurrent UTI (RUTI) can damage renal function, and even the first episode of acute pyelonephritis can result in renal scarring^3^. RUTI significantly increases healthcare costs and has a detrimental impact on patients' quality of life^4,5^. *Escherichia coli* is the leading pathogen responsible for both sporadic and recurrent UTI (RUTI)^6^. Our previous study demonstrated that there are different roles of host and bacterial factors in *E. coli* extra-intestinal infections, including urinary tract infection^7^.

Artificial intelligence (AI) methods have been widely used in medical practice and health care. AI programs can perform clinical classification, disease diagnosis, and treatment recommendation, etc.^8,9^. Based on clinical symptoms, laboratory findings and ultrasound, development of AI models [decision tree (DT), support vector machine, random forest (RF), and artificial neural network] could support the diagnosis of UTI with complex symptoms with high accuracy (> 93%), sensitivity (> 95%), and specificity (> 85%)^10^. The predictive model built using different machine learning tools and cloud platform may serve as a useful support tool for physicians to treat hospitalized patients at high risk of multidrug resistant UTI^11^. However, there is no literature published on prediction of RUTI based on artificial intelligence methods. The aim of this study was to predict the development of RUTI related to *E. coli* using machine learning. Additionally, we built the prediction models that included the important clinical host characteristics and the bacterial characteristics (phylogenicity, virulence, and profile of antimicrobial susceptibility).

## Methods

### Patient population and data collection

This is a single-center retrospective cohort study. The study enrolled patients aged 20 years or above who presented with symptoms of UTI in emergency department (ED) or outpatient clinics of National Cheng Kung University Hospital (NCKUH), a tertiary-care, 1200-bed teaching hospital in Taiwan, between August 2009 and December 2010. Included patients were diagnosed to have UTI related to *E. coli* based on the clinical features, physical examination, urine analysis, urine culture, and/or imaging studies. RUTI is defined as: patients had 2 or more infections in 6 months or 3 or more in 12 months during the study period^12^. Patients were admitted if they have severe illness, upper UTI, or hemodynamic instability. This study was reviewed and approved by the Institutional Review Board of National Cheng Kung University Hospital, Tainan, Taiwan (B-ER-109–565). The informed consent was waived by the institutional review board who approved the study. We confirm that all methods were performed in accordance with the relevant guidelines and regulations.

The dataset of this study had different qualitative inputs obtained from the characteristics of patients and bacteria. Patient and bacterial characteristic factors included age, gender, history and frequency of urinary tract infection or hospitalization within 2 years, visit at outpatient department or ED, diabetes mellitus, malignancy with exclusion of urogenital cancer, autoimmune disease (systemic lupus erythematosus and rheumatoid arthritis), liver cirrhosis, indwelling Foley catheter, obstructive uropathy, urolithiasis, urogenital malignancy, neurogenic bladder, end stage renal disease (undergoing hemodialysis and peritoneal dialysis therapy), organ transplantation, stroke, upper or lower UTI, serum creatinine, white blood cell count in blood, red blood cell count and white blood cell count in urine analysis, length of hospital stay, profiles of *E. coli* antimicrobial susceptibility tests, and bacterial phylogenicity and virulence genes.

There were 2 stages for development of prediction models for RUTI. The first stage was a scenario at the clinical visit (outpatient department or ED) for UTI with 963 patients, where RUTI and non RUTI patient numbers were 136 and 827, respectively. The second stage was a scenario after hospitalization with a complete survey of UTI with 809 patients, where RUTI and non RUTI patient numbers were 112 and 687, respectively. For the first stage analysis, the patient characteristic factors included medical history, clinical features, and laboratory tests. For the second stage analysis, bacterial characteristic factors, profiles of antimicrobial susceptibility tests, bacterial phylogenicity, and virulence genes were also used. The detail tables of the factors are given in the Result session.

In the data preprocess, the k-nearest neighbors algorithm (*k* = 10) was applied to deal with the missing data imputation for the second stage data. In the model training, an upsampling technique was applied to the dataset with a disproportionate ratio of observations in each group.

### Model training and validation

The fivefold cross-validation method was used to evaluate the performance of the models. Dataset was divided into 5 folds randomly with equal size. The first fold was treated as a validation set, and the models were fit on the remaining 4 folds. The mean validation accuracy was computed on the observations in the held-out fold. This procedure was repeated 5 times. A different group of observations was treated as a validation set at each time. This process resulted in 5 estimated test accuracies, ${\mathrm{Acc}}_{1}$, ${\mathrm{Acc}}_{2}$, …, ${\mathrm{Acc}}_{5}$. The fivefold CV estimate was computed by averaging these values, $\mathrm{CV}=\frac{1}{n}\sum_{i=1}^{5}{\mathrm{Acc}}_{\mathrm{i}}$, where ${\mathrm{Acc}}_{\mathrm{i}}=\mathrm{I}({y}_{i}={\widehat{y}}_{i})$. The standard deviation was also calculated from the 5 estimated test accuracies.

The accuracy, sensibility, and specificity the models will be reported. True positive and true negative are the classification results that correctly indicate the presence and absence of a condition, respectively. Accuracy refers to the ratio of the number of true positive and true negative patients to the total number of patients. Sensitivity (true positive rate) refers to the ratio of the number of true positive patients to the number of positive patients. Specificity (true negative rate) refers to the ratio of the number of true negative patients to the number of negative patients.

### Learning models and statistical tests

We developed models for RUTI prediction using 3 machine learning algorithms: logistic regression (LR), DT, and RF.

LR is an extension of traditional regression wherein a set of independent factors is usually used to model a binary outcome. Logistic regression is an appropriate method for this study to model the dichotomous variable of patients with and without RUTIs. Logistic regression builds the model to predict the odds of an event’s occurrence (RUTI) using weights to maximize the likelihood of reproducing the data^13,14^.

Decision trees are tree-like structures that start from root nodes and end with leaf nodes. The model has several branches consisting of different factors, and the leaf node on each branch represents a class or a kind of class distribution. Decision trees describe the relationship among factors and the relative importance of factors. Each branch of the tree provides a decision rule for the classification by the factors. This method uses recursive data separation to construct a tree, by repeatedly splitting the branches into subgroups until splitting no longer adds any information to the predictions. Mathematical algorithms are used to identify a factor and corresponding threshold that splits the input observation into two or more subgroups. The Gini index is a widely used split criterion in DTs, a statistical measure of distribution to evaluate how mixed the classes are split into two groups^13^.

RF constructed a multitude of decision trees at training time and output the class that was the mode of the classes. RF split random sample of factors as split candidate from the whole factors in each time while it built these decision trees. The split was allowed to use only one of those factors. A fresh sample of factors was taken at each split, and typically the number of factors considered at each split was approximately equal to the square root of the total number of factors^9^. The prediction power of RF usually is higher than DT. However, the decision rules are not tractable in the RF model.

Several statistical tests were implemented to evaluate the relation between the factors and the RUTI. The Chi-square test or Fisher’s exact test (two-tailed) was used for the comparison of categorical factors, whereas the Wilcoxon rank-sum test or Pearson’s Chi-squared test was used for the comparison of continuous factors between groups. A *P* value < 0.05 was considered to be statistically significant.

## Results

A total of 963 *E. coli* UTI patients from NCKUH were included, 14.2% of them had *E. coli* RUTI. All the 137 RUTI patients included in this study had RUTI caused by *E. coli*, 74 patients (54%) had 2 episodes of UTI within 6 months and 63 patients (46%) had 3 episodes of UTI within 12 months. All these episodes of *E. coli* related RUTI in this study were reinfection (recurrence of UTI with the same organisms in more than 2 weeks). The duration of antibiotic treatment varied from 3 to 14 days, and the antibiotic regimens included empirical antibiotic therapy and definitive antibiotic therapy according to the antimicrobial susceptibility test. The patient characteristics related to UTI and RUTI caused by *E. coli* are shown in Table 1. The median age was 67 and 75 years for patients with UTI and RUTI, respectively. Compared to the UTI group, patients with RUTI had an older age, a greater prevalence of diabetes mellitus, liver cirrhosis, indwelling Foley catheter, neurogenic bladder, more frequent hospitalization/emergency department (ED) visit/UTI within 2 years and any UTI symptom, and a worse renal function (Table 1).

**Table 1:** Patient characteristics related to UTI and RUTI (sample size = 963) used in the first stage analysis. The name in the parentheses represents the label of the factor used in the machine learning models. Data are presented with median (interquartile range) or number (percentage). Abbreviations: UTI, urinary tract infection; RUTI, recurrent urinary tract infection; ED, emergency department; WBC, white blood cell; RBC, red blood cell; HPF, high power field.

Characteristic	UTI (*n* = 826)	RUTI (n = 137)	*P* value
Age (year)	67 (45–78)	75 (62–81)	< 0.0001
Gender (male)	208 (25)	35 (26)	0.9157
Place of urine sample collection (ED) (Place_of_collection)	781 (95)	123 (90)	0.0513
Diabetes mellitus (Dis1)	230 (28)	63 (46)	< 0.0001
Malignancy, exclusion of urogenital malignancy (Dis2)	117 (14)	19 (14)	0.9999
Autoimmune disease (Dis3)	15 (2)	1 (1)	0.7146
Liver cirrhosis (Dis4)	24 (3)	12 (9)	0.0025
Indwelling Foley catheter (Dis5)	35 (4)	13 (9)	0.0172
Obstructive uropathy (Dis6)	100 (12)	23 (17)	0.1299
Urolithiasis (Dis7)	20 (2)	4 (3)	0.7653
Urogenital malignancy (Dis8)	19 (2)	6 (4)	0.1519
Neurogenic bladder (Dis9)	35 (5)	22 (16)	< 0.0001
Disease group (four_disease_group)	154 (18)	51 (37)	< 0.0001
End stage renal disease (Dis10)	18 (2)	4 (3)	0.5394
Transplantation (Dis11)	5 (1)	1 (1)	0.9999
Stroke (Dis12)	65 (8)	13 (9)	0.5004
Frequency of hospitalization within 2 years (Pre_hos_2y)	0 (0–2)	1 (0–3)	< 0.0001
Frequency of ED visit within 2 years (Pre_UTI_ER_2y)	0 (0–0)	0 (0–1)	0.0004
Frequency of UTI within 2 years (Pre_UTI_hos_2y)	0 (0–1)	0 (0–2)	< 0.0001
**Any UTI symptom**	441 (53)	59 (43)	0.0268
Fever (UTI_symptom1)	375 (45)	50 (36)	0.0629
Dysuria (UTI_symptom2)	74 (9)	12 (9)	0.9999
Painful urination (UTI_symptom3)	0 (0)	1 (1)	0.1423
Frequency (UTI_symptom4)	72 (9)	7 (5)	0.1800
Burning sensation (UTI_symptom5)	31 (4)	4 (3)	0.8070
Low abdominal pain (UTI_symptom6)	15 (2)	1 (1)	0.7146
Flank/back pain (UTI_symptom7)	63 (8)	5 (4)	0.1053
Gross hematuria (UTI_symptom8)	36 (4)	5 (4)	0.8228
Serum creatinine (mg/dL)	0.8 (0.6–1.2)	0.96 (0.6–2.0)	0.0101
Peak blood WBC count (10^9^/L) (BloodWBC)	10.9 (8.1–14.4)	10.2 (7.3–13.4)	0.0761
Urinary bacterial count (0 ~ 4) (UBact)	2 (1–3)	2 (1–3)	0.4160
Urinary WBC/HPF (UWBC_level)	52 (15–178)	41 (12–122)	0.3491
Urinary RBC/HPF (URBC_level)	5 (1–20)	5 (1–11)	0.3540

The bacterial characteristic factors (phylogenicity, virulence genes, and antimicrobial susceptibility) related to UTI and RUTI are shown in Tables 2 and 3, respectively. Compared to those in the UTI group, *E. coli* isolates derived from the RUTI group had a lower prevalence of *papG II*, *usp*, *ompT*, and *sat* genes, and a higher prevalence of antimicrobial resistance in several antibiotics (including cefazolin, cefuroxime, cefixime, and levofloxacin).

**Table 2:** Bacterial characteristics related to UTI and RUTI (sample size = 809) used in the second stage analysis. The name in the parentheses represents the label of the factor used in the machine learning models. Data are presented with number (percentage). Abbreviations: UTI, urinary tract infection; RUTI, recurrent urinary tract infection.

Characteristic	UTI (*n* = 697)	RUTI (*n* = 112)	*P* value
**Phylogenicity** (Gene17)	*n* = 436	*n* = 85	0.1308
A (1)	57 (13)	12 (14)	
B1 (2)	39 (9)	11 (13)	
B2 (3)	251 (58)	38 (45)	
D (4)	89 (20)	24 (28)	
**Virulence gene**			
*papG II* (Gene2)	*n* = 462	*n* = 86	0.0004
	154 (33)	11 (13)	
*papG III* (Gene3)	*n* = 462	*n* = 86	0.8645
	62 (13)	12 (14)	
*papG II or III*	*n* = 462	*n* = 86	0.0028
	197 (43)	22 (26)	
*sfa* (Gene4)	*n* = 462	*n* = 86	0.7995
	25 (5)	5 (6)	
*foc* (Gene5)	*n* = 462	*n* = 86	0.0674
	36 (8)	2 (2)	
*cnf1* (Gene6)	*n* = 462	*n* = 86	0.2505
	74 (16)	9 (10)	
*aer* (Gene7)	*n* = 462	*n* = 86	0.6245
	299 (65)	53 (62)	
*usp* (Gene8)	*n* = 462	*n* = 86	0.0234
	282 (61)	41 (48)	
*iha* (Gene9)	*n* = 461	*n *= 86	0.0697
	183 (40)	25 (29)	
*ompT* (Gene10)	*n* = 462	*n* = 86	0.0169
	346 (75)	53 (62)	
*afa* (Gene11)	*n* = 462	*n* = 86	0.6344
	272 (59)	48 (56)	
*iRONE* (Gene12)	*n* = 462	*n* = 86	0.0660
	173 (37)	23 (27)	
*fimH* (Gene13)	*n* = 462	*n* = 86	0.3323
	436 (94)	79 (92)	
*hlyA* (Gene14)	*n* = 462	*n* = 86	0.0782
	98 (21)	11 (13)	
*sat* (Gene15)	*n* = 428	*n* = 84	0.0341
	159 (37)	21 (25)	
*neuA* (Gene16)	*n* = 462	*n* = 86	0.1741
	120 (26)	16 (19)

**Table 3:** Antimicrobial susceptibility of bacterial pathogens related to UTI and RUTI (sample size = 809) used in the second stage analysis. The name in the parentheses represents the label of the factor used in the machine learning models. Data are presented with number (percentage). Abbreviations: UTI, urinary tract infection; RUTI, recurrent urinary tract infection. S, susceptible; I, intermediate; R, resistant.

Antimicrobial susceptibility	UTI (*n* = 697)	RUTI (*n* = 112)	*P* value
**Ampicillin (Anti4)**	*n* = 638	*n* = 98	0.2392
S	126 (20)	13 (13)	
I	3 (0)	0 (0)	
R	509 (80)	85 (87)	
**Amoxicillin/clavulanic acid (Anti2)**	*n* = 454	*n* = 69	0.0838
S	277 (61)	35 (50)	
I	52 (11)	6 (9)	
R	125 (28)	28 (41)	
**Cefazolin (Anti5)**	*n* = 638	*n* = 98	0.0003
S	407 (64)	44 (45)	
I	18 (3)	1 (1)	
R	213 (33)	53 (54)	
**Cefuroxime (Anti14)**	*n* = 628	*n* = 96	0.0058
S	430 (68)	50 (52)	
I	22 (4)	4 (4)	
R	176 (28)	42 (44)	
**Cefmetazole (Anti8)**	*n* = 450	*n* = 68	0.4653
S	350 (77)	49 (72)	
I	16 (4)	2 (3)	
R	84 (19)	17 (25)	
**Cefixime (Anti7)**	*n* = 637	*n* = 98	0.0004
S	435 (68)	45 (46)	
I	5 (1)	1 (1)	
R	197 (31)	52 (53)	
**Ceftriaxone (Anti13)**	*n* = 451	*n* = 68	0.0897
S	311 (69)	38 (56)	
I	62 (14)	12 (18)	
R	78 (17)	18 (26)	
**Cefpirome (Anti11)**	*n* = 637	*n* = 98	0.0807
S	559 (88)	78 (80)	
I	14 (2)	3 (3)	
R	64 (10)	17 (17)	
**Ertapenem (Anti19)**	*n* = 623	*n* = 95	0.1509
S	621 (100)	93 (98)	
I	0 (0)	0 (0)	
R	2 (0)	2 (2)	
**Gentamicin (Anti23)**	*n* = 636	*n* = 98	0.0994
S	420 (66)	54 (55)	
I	13 (2)	2 (2)	
R	203 (32)	42 (43)	
**Amikacin (Anti1)**	*n* = 634	*n* = 97	0.3417
S	622 (98)	94 (97)	
I	8 (1)	1 (1)	
R	4 (1)	2 (2)	
**Levofloxacin (Anti25)**	*n* = 636	*n* = 98	0.0010
S	403 (63)	43 (44)	
I	6 (1)	2 (2)	
R	227 (36)	53 (54)	
**Co-trimoxazole (Anti18)**	*n* = 625	*n* = 95	0.0840
S	281 (45)	33 (35)	
I	7 (1)	0 (0)	
R	337 (54)	62 (65)	

### First stage: predict the development of *E. coli* RUTI in the clinical visit (sample size = 963)

The analysis results suggested RF model was better than the LR and DT model for RUTI prediction in the clinical visit. The 32 factors considered in the models for the first stage were age, gender, comorbidities (Dis1 ~ Dis12), UTI symptoms (UTI_symptom1 ~ UTI_symptom8), serum creatinine, frequency of hospitalization/emergency department (ED) visit/UTI within 2 years (Pre_hos_2y, Pre_UTI_ER_2y, Pre_UTI_hos_2y), urinary red blood cell/HPF (URBC_level), urinary white blood cell (WBC)/high power field (HPF) (UWBC_level), urinary bacterial count (UBact), peak blood WBC count (BloodWBC), place (outpatient or ED) of urine sample collection (Place_of_collection), and disease group (four_disease_group). These factors are labeled in Table 1.

URBC_level and UWBC_level represent the rescaled level of the URBC and UWBC with values from 0 to 4 and from 1 to 4, respectively. The values 0, 1, 2, 3, and 4 of the URBC_level and UWBC_level corresponded to the ranges 0, 1 ~ 10, 11 ~ 100, 101 ~ 1000, and greater than 1000 per HPF, respectively. Place_of_collection indicates the place of urine sample collection, including outpatient clinic and ED. A new factor called four_disease_group was defined for RUTI prediction with value 0 or 1. We set four_disease_group value to 1 when one of the following diseases with anatomical or functional defect of urinary tract is present: indwelling Foley catheter (Dis5), obstructive uropathy (Dis6), urolithiasis (Dis7), and neurogenic bladder (Dis9). We would like to confirm the relation of four_disease_group with RUTI.

Regarding the validation results of fitted models to predict the development of RUTI in the clinical visit, Table 4 shows that the mean validation accuracy of RF is 0.700 which is higher than the results of LR and DT. The mean validation sensitivity and specificity of RF are 0.626 and 0.712, respectively. The standard deviations of estimated validation accuracy, sensibility, and specificity are 0.039, 0.131, and 0.046, respectively, which support the stability of RF model prediction. Note that the RUTI rate is only 136/963 = 0.138 which is relatively low for the observed samples. A naïve model would predict non of the patients to have RUTI with a high accuracy 827/963 = 0.862. However, such prediction will lead to a very poor sensitivity with value 0. The RF model avoided such serious bias and provided a balance prediction capability in both sensitivity and specificity. The key technique in the RF model training is the usage of upsampling.

**Table 4:** Comparison of the performance in RUTI prediction models in the clinical visit through fivefold cross validation (sample size = 963). Abbreviation: RUTI, recurrent urinary tract infection.

Algorithm	Accuracy		Sensitivity		Specificity	
	Mean	Standard deviation	Mean	Standard deviation	Mean	Standard deviation
Logistic regression	0.604	0.044	0.648	0.117	0.597	0.044
Decision tree	0.654	0.020	0.618	0.058	0.660	0.023
Random forest	0.700	0.039	0.626	0.131	0.712	0.046

**Figure 1:**
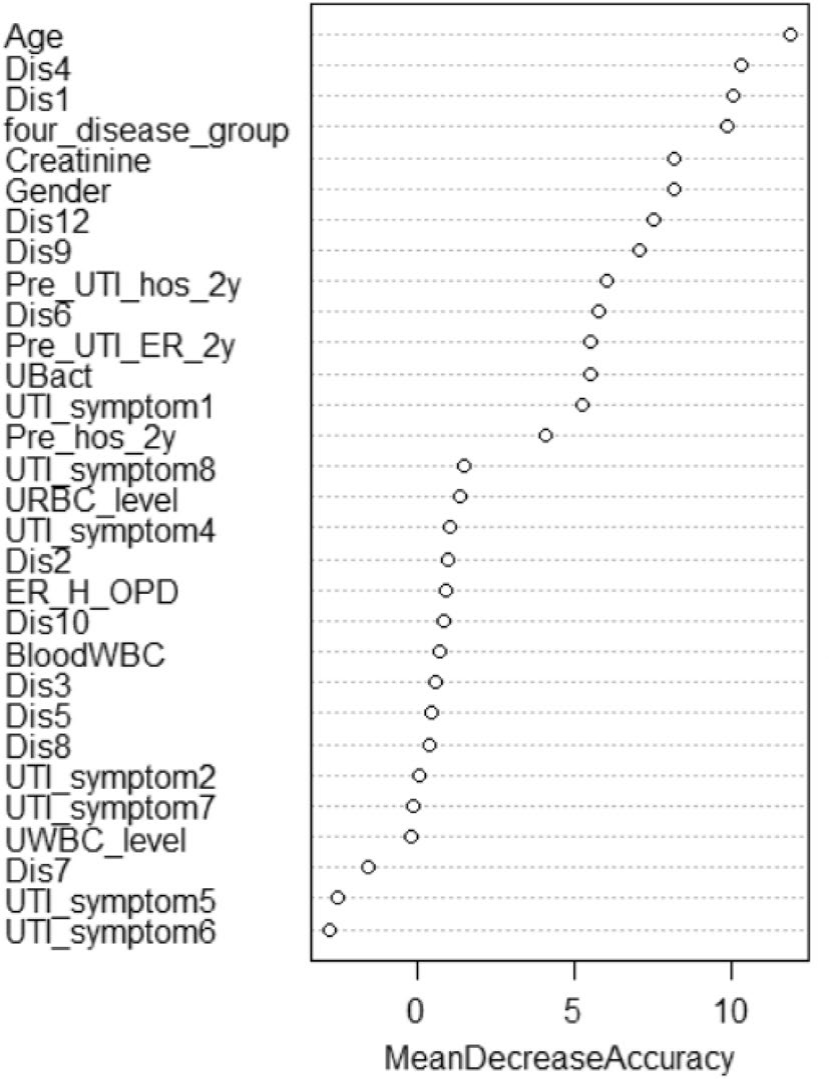
Variable importance plot of the first stage RF analysis in percentage of mean decrease accuracy for the factors. It shows that age, cirrhosis (Dis4), diabetes mellitus (Dis1), and disease group (four_disease_group) are the most important 4 factors to predict recurrence in the clinical visit (sample size = 963).

Variable importance in RF is evaluated by the mean decrease of accuracy in predictions on the out of bag samples when a given variable is excluded from the model. For example, if the age is taken away, the model prediction will reduce the accuracy rate by 11.9%. Figure 1 is the variable importance plot of the RF analysis and shows that age, cirrhosis (Dis4), diabetes mellitus (Dis1), and disease group (four_disease_group) are the most important factors to predict recurrence of UTI in the clinical visit. Each of the 4 factors contributed around 10% prediction accuracy in the RF model.

A DT model is able to construct the decision rules for RUTI classification and provides the order of importance of the factors at the same time. Table 4 shows that the mean validation accuracy, sensitivity, and specificity of DT model are 0.654, 0.618, and 0.660, respectively. Although the validation accuracy of the DT is less than the values of the RF model, the results of DT model has its own edge in decision rule construction.

**Figure 2:**
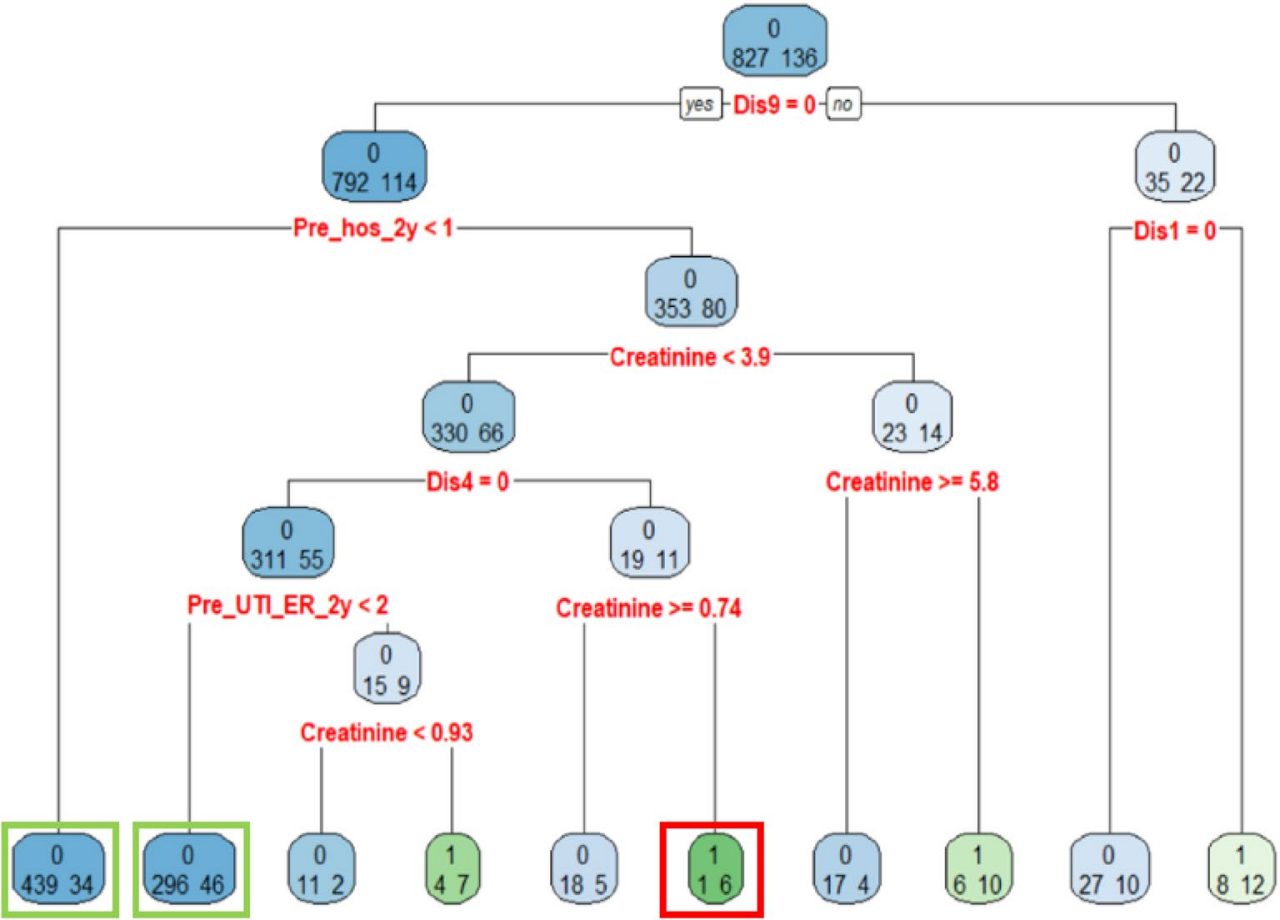
The decision rules of the DT analysis for development of RUTI in the clinical visit. (sample size = 963). The 2 green boxes and 1 red box indicate the nodes of the decision rules with an accuracy rate higher than 0.85 and 0.70 for non RUTI and RUTI classification, respectively.

To obtain more insight on the RUTI factors in the clinical visit, one can check on Fig. 2 which is the decision rules of the DT model built from all the 963 patients. The purpose of building a DT model with all collected data is to construct the decision rules for RUTI classification. In a DT model, when the patients satisfy the node's condition, the patients will be allocated to the left path of the node, otherwise the patients will be allocated to the right path of the node. The classification accuracy of this tree is 0.88, and the sensitivity and specificity are 0.26 and 0.98, respectively. Although the sensitivity is low due to the unbalanced rates of RUTI and UTI in the DT model, there are several valuable rules for RUTI classification. The 2 green boxes and 1 red box in Fig. 2 indicate the nodes of the decision rules with a accuracy rate higher than 0.85 and 0.70 for non RUTI and RUTI classification, respectively. The three decision rules are:

1. When the factor states of a patient are without neurogenic bladder (Dis9 = 0) and without hospitalized within 2 years (Pre_hos_2y < 1), this rule claims that the patient will have no RUTI with classification accuracy 439/(439 + 34) = 0.92.
2. When the factor states of a patient are without neurogenic bladder (Dis9 = 0), with previous hospitalization at least one time within 2 years (Pre_hos_2y >  = 1), with serum creatinine less than 0.93 mg/dL (creatinine < 0.93), without cirrhosis (Dis4 = 0), and previous ER for UTI less than two times within 2 years (Pre_UTI_ER_2y < 2), this rule claims that the patient will have no RUTI with classification accuracy 296/(296 + 46) = 0.86.
3. When the factor states of a patient are without neurogenic bladder (Dis9 = 0), with previous hospitalization at least one time within 2 years (Pre_hos_2y >  = 1), with serum creatinine in the range between 0.74 and 3.9 mg/dL (0.74 < creatine < 3.9), with cirrhosis (Dis4 = 1), this rule claims that the patient will have RUTI with classification accuracy 6/(6 + 1) = 0.85.

### Second stage: predict the development of *E. coli* RUTI after hospitalization for *E. coli* UTI (sample size = 809)

The analysis results suggested RF model was better than the LR and DT model for RUTI prediction after hospitalization. The 62 factors considered in the models for the second stage not only contain the 32 factors used in the first stage analysis, but also include phylogenicity, 16 virulence genes, 11 antimicrobial susceptibility, Bacterial_Name, UTI_pos, Hospitalday, and Place_of_collection. The genes and antimicrobial are labeled in Table 2. Bacterial_name indicates *Escherichia coli* with or without extended spectrum β-lactamase (ESBL). UTI_pos represents the location of urinary tract infection. Hospital_day gives the length (day) of hospital stay. Place_of_collection records the place of sample collection at ER, hospital, or outpatient clinic.

Regarding the validation results of refitted models to predict the development of RUTI after hospitalization, Table 5 shows that the mean validation accuracy of RF is 0.709 which is higher than the results of LR and DT. The mean validation sensitivity and specificity of RF are 0.620 and 0.722, respectively. The standard deviations of estimated validation accuracy, sensibility, and specificity are 0.047, 0.057, and 0.058, respectively, which support the stability of RF model prediction. Note that the RUTI rate is only 112/809 = 0.138 which is relatively low for the observed samples. A naïve model would predict non of the patients to have RUTI with a high accuracy 697/809 = 0.862. However, such prediction will lead to a very poor sensitivity with value 0. The RF model avoided such serious bias and provided a balance prediction capability in both sensitivity and specificity.

**Table 5:** Comparison of the performance in RUTI prediction models after hospitalization for UTI through fivefold cross validation (sample size = 809). Abbreviations: UTI, urinary tract infection; RUTI, recurrent urinary tract infection.

Algorithm	Accuracy		Sensitivity		Specificity	
	Mean	Standard deviation	Mean	Standard deviation	Mean	Standard deviation
Logistic regression	0.604	0.026	0.590	0.065	0.605	0.034
Decision tree	0.635	0.052	0.600	0.061	0.640	0.057
Random forest	0.709	0.047	0.620	0.057	0.722	0.058

**Figure 3:**
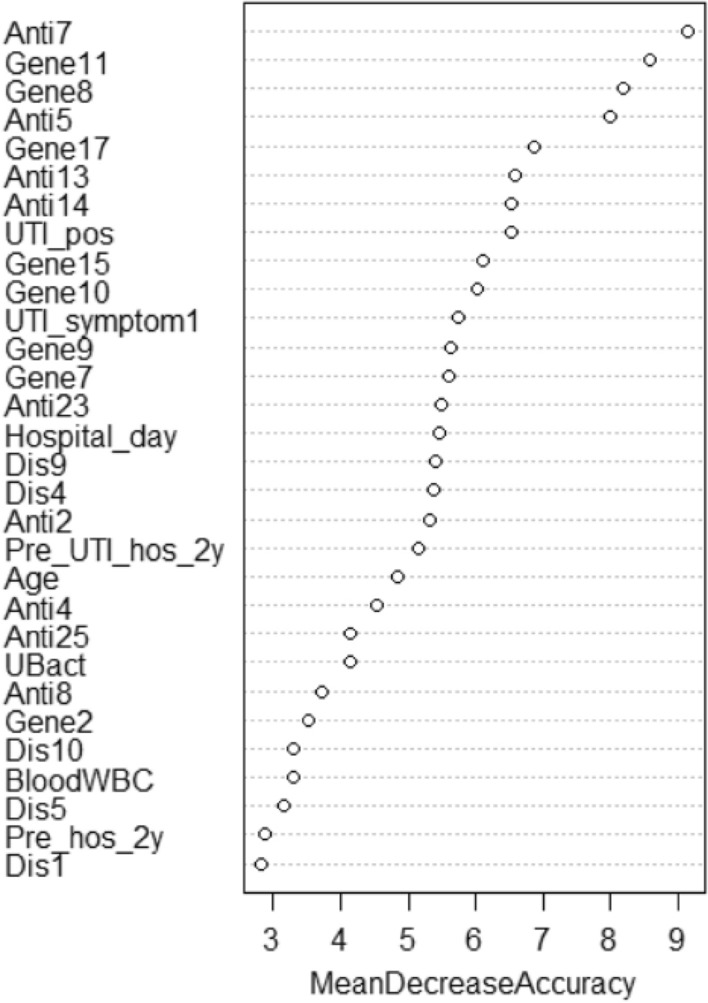
Variable importance plot of the second stage RF analysis in percentage of mean decrease accuracy for the factors. It shows that cefixime (Anti7), *afa* (Gene11), *usp* (Gene8), and cefazolin (Anti5) are important factors to predict recurrence after hospitalization (sample size = 809).

Variable importance plot shows that based upon the mean decrease of accuracy in predictions on the out of bag samples when a given variable is excluded from the model. For example, if the cefixime (Anti7) is taken away, the model prediction will reduce the accuracy rate by 9.14%. Figure 3 is the variable importance plot of the RF analysis and shows that cefixime (Anti7), *afa* (Gene11), *usp* (Gene8), and cefazolin (Anti5) are important factors to predict recurrence after hospitalization. Each of the 4 factors contributed more than 8% prediction accuracy in the RF model.

**Figure 4:**
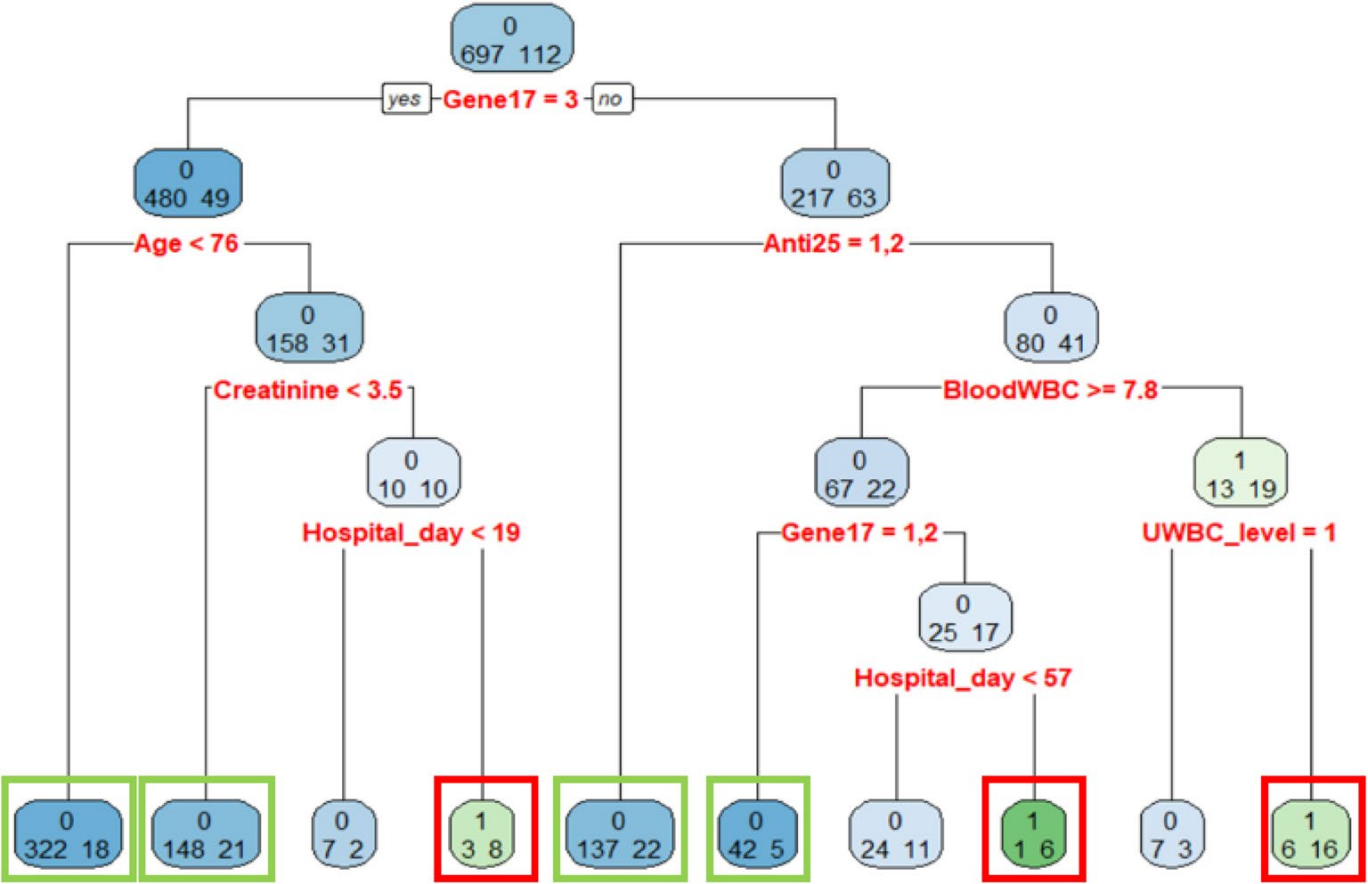
The decision rules of the DT analysis for development of RUTI after hospitalization. The 4 green boxes and 3 red boxes indicate the nodes of the decision rules with an accuracy rate higher than 0.85 and 0.70 for non RUTI and RUTI classification, respectively (sample size = 809).

To obtain more insight on the RUTI factors after hospitalization, one can check on Fig. 4 which is the decision rules of the DT model built from all the 803 patients. The classification accuracy of this tree is 0.89, and the sensitivity and specificity are 0.27 and 0.99, respectively. Although the sensitivity is low due to the unbalanced rates of RUTI and UTI in the DT model, there are several valuable rues for RUTI classification. The 4 green boxes and 3 red boxes in Fig. 4 indicate the nodes of the decision rules with an accuracy rate higher than 0.85 and 0.70 for non RUTI and RUTI classification, respectively. The 7 decision rules are:

1. When the factor states of a patient are bacterial phylogenetic group B2 (Gene17 = 3) and the age less than 76 years old (Age < 76), this rule claims that the patient will have no RUTI with classification accuracy 322/(322 + 18) = 0.94.
2. When the factor states of a patient are bacterial phylogenetic group B2 (Gene17 = 3), the age over 76 years old (Age $\ge$ 76), and serum creatinine less than 3.5 mg/dL (creatinine < 3.5), this rule claims that the patient will have no RUTI with classification accuracy 148/(148 + 21) = 0.87.
3. When the factor states of a patient are bacterial phylogenetic group B2 (Gene17 = 3), the age over 76 years old (Age $\ge$ 76), serum creatinine less than 3.5 mg/dL (creatinine $\ge$ 3.5), and more than 19 days of hospital stay (Hospital_day $\ge$ 19), this rule claims that the patient will have RUTI with classification accuracy 8/(3 + 8) = 0.72.
4. When the factor states of a patient are non-group B2 in bacterial phylogenicity (Gene17 $\ne$ 3) and S or I type in levofloxacin susceptibility (Anti25 = 1, 2), this rule claims that the patient will have no RUTI with classification accuracy 137/(137 + 22) = 0.86.
5. When the factor states of a patient are non-group B2 in bacterial phylogenicity (Gene17 $\ne$ 3), R type in levofloxacin susceptibility (Anti25 = 3), bloodWBC more than 7.8 (bloodWBC $\ge$ 7.8), and group A or B1 in bacterial phylogenicity (Gene17 = 1, 2), this rule claims that the patient will have no RUTI with classification accuracy 42/(42 + 5) = 0.89.
6. When the factor states of a patient are non-group B2 in bacterial phylogenicity (Gene17 $\ne$ 3), R type in levofloxacin susceptibility (Anti25 = 3), bloodWBC more than 7.8 (bloodWBC $\ge$ 7.8), group D in phylogenicity (Gene17 = 4), and more than 57 days of hospital stay (Hospital_day $\ge$ 57), this rule claims that the patient will have RUTI with classification accuracy 6/(6 + 1) = 0.85.
7. When the factor states of a patient are non-group B2 in bacterial phylogenicity (Gene17 $\ne$ 3), R type in levofloxacin susceptibility (Anti25 = 3), bloodWBC less than 7.8 (bloodWBC < 7.8), and the value of UWBC more than 10 (UWBC_level $\ne$ 1), this rule claims that the patient will have RUTI with classification accuracy 16/(6 + 16) = 0.72.

## Discussion

UTI is one of the most common infectious diseases in both women and men, and RUTI can cause disability and subsequent morbidity, especially in patients at risk of RUTI, and place a large burden on medical care. There have been several studies presenting the prediction and discovery of UTI based on artificial intelligence methods^10,15–17^. However, there have been scarce reports dealing with the prediction of RUTI using machine learning models. Our study investigated RUTI caused by the most common pathogen, *E. coli*, and used machine learning models to predict the development of RUTI. Based on the single uropathogen, bacterial characteristics and antimicrobial susceptibility of *E. coli* could be included for analyses in addition to host characteristics, clinical features and laboratory tests in the prediction models. We successfully developed and validated machine learning models which showed good accuracy in predicting the development of RUTI, and RF could provide a better accuracy than LR and DT in the clinical visit and after hospitalization for UTI.

Gadalla et al. explored 17 clinical and 42 immunological potential predictors for women with uncomplicated UTI, and reported that urine cloudiness was the best clinical predictor to rule out and rule in UTI^15^. Ozkan et al. demonstrated that artificial neural network had the highest accuracy of 98.3% for UTI diagnosis compared to the other models (DT, support vector machine, and RF models; 93.22%, 96.61%, 96.61%, respectively) in 59 patients (35 female and 24 male)^10^. Their ANN model only needs 2 symptoms and urine erythrocyte to get the same diagnosis with such accuracy. The classification target was aimed at the separation of cystitis and urethritis where the different symptoms would be revealed in the full urine analysis and the renal and bladder ultrasound. This was the possible reason to obtain the high accuracy of UTI diagnosis. Chen et al. compared the neural network and LR analysis to predict the probability of UTI caused by cystoscopy in 1647 patients with occurrence of UTI in 147 cases. The LR model had an accuracy of 91%, sensitivity of 2% and specificity of 99%, and neural network model had an accuracy of 85%, sensitivity of 80%, and specificity of 88%^16^. No cross validation procedure was reported in their analysis. The stability of the accuracy, sensitivity, and specificity values were not explored. Our study demonstrated that RF model provided a better model than LR and DT in predicting the development of RUTI in accuracy and specificity. Application of the prediction models need to balance the sensitivity and specificity in different scenarios by physicians. For example, a model with higher sensitivity could benefit the decision making with aggressive intervention for patients with important risk factors in the model. Although the accuracy was not very high in both scenarios (0.700 and 0.709, respectively) compared to those reported in UTI prediction in literature, we believe that it is because our study design focused on RUTI caused by single uropathogen (*E. coli*), but not all uropathogens, in order to include the bacterial characteristics (phylogenicity, virulence, and profile of antimicrobial susceptibility) for further analysis in stage 2. Besides, in the literature dealing with UTI prediction using artificial intelligence, UTI caused by all pathogens were included and RUTI was not excluded from the UTI events in the dataset, which may have impact on the prediction values.

In the clinical visit (stage 1), there were several factors showing significant differences between UTI and RUTI groups (older age, greater prevalence of comorbidities, and higher frequency of hospitalization/ED visit/UTI within 2 years in the RUTI group). Variable importance plot of RF analysis revealed similar results and that age, cirrhosis, diabetes mellitus, and disease group were the most important 4 factors to predict RUTI in the clinical visit. These are also well-known risk factors for UTI. Although there was no improvement in the prediction accuracy after including the bacterial virulence and antimicrobial profile in the prediction model in stage 2, variable importance plot of RF analysis showed that uropathogenic *E. coli* strains resistance to cefixime and cefazolin as well as exhibiting *afa* and *usp* genes were important factors to predict RUTI after hospitalization for UTI. These important factors to predict RUTI in stage 2 were different from those in stage 1, and the importance of host factors in the variable importance plot of RF in stage 1 was replaced by bacterial factors in stage 2. The inclusion of bacterial characteristics (phylogenicity, virulence, and profile of antimicrobial susceptibility) in prediction models seemed only to increase the specificity in predicting the development of RUTI. Our study also demonstrated the role of decision rules derived from the DT analysis in evaluating the risk of developing RUTI, which could increase the accuracy in certain subgroup patients in different scenarios. For example, if a patient visits the outpatient clinic/ED without a history of neurogenic bladder or hospitalization within 2 years, the patient will have no RUTI with a classification accuracy rate of 0.92.

There are several limitations in this study. First, this was a single-center study with a retrospective design and a relatively small sample size. A multicenter prospective study with a larger sample size will be needed to confirm our results. Second, not all important characteristics of patients and bacteria were included for analyses. Third, not all models of machine learning were applied for prediction of RUTI. Fourth, determination of bacterial phylogenicity and/or virulence genes is expensive and needs more complicated procedure for tests.

In conclusion, this study provides good machine learning models in predicting the development of RUTI caused by *E. coli*, the most common uropathogen, in 2 clinic stages (in the clinical visit and after hospitalization for UTI). RF could provide a better accuracy than LR and DT in both stages, and decision rules derived from the DT analysis could provide high accuracy in certain subgroup patients in different clinical scenarios. Our study also demonstrated that host and bacterial characteristics made important contribution to the development of RUTI in the prediction models in 2 scenarios*,* respectively. For patients at an increased risk of *E. coli* RUTI, it is important for physicians to improve the functional/anatomical urinary tract abnormalities and immunocompromised conditions of patients in order to prevent the development of RUTI.

## Data Availability

The datasets generated during and/or analyzed during the current study are available from the corresponding author on reasonable request.
